# Prognostic value of the baseline 18F-FDG PET/CT metabolic tumour volume (MTV) and further stratification in low-intermediate (L-I) and high-intermediate (H-I) risk NCCNIPI subgroup by MTV in DLBCL MTV predict prognosis in DLBCL

**DOI:** 10.1007/s12149-020-01531-1

**Published:** 2020-10-01

**Authors:** Peng Zhao, Tao Yu, Zheng Pan

**Affiliations:** 1 Cancer Hospital of China Medical University, Liaoning Cancer Hospital &Institute, Shenyang, Liaoning People’s Republic of China; 2 Cancer Hospital of China Medical University, Liaoning Cancer Hospital &Institute, No. 44 Xiaoheyan Road, Dadong District, Shenyang, Liaoning 110042 People’s Republic of China

**Keywords:** PET/CT, Diffuse large B-cell lymphoma, Metabolic tumour volume, Prognosis

## Abstract

**Introduction:**

In the era of rituximab, the NCCNIPI is widely used in clinical practice as a tool for the prognosis and risk stratification of diffuse large B-cell lymphoma (DLBCL). In recent years, FDG PET/CT has also shown unique prognostic value. We try to further confirm the prognostic role of metabolic parameters in the overall and subgroups patients.

**Methods:**

We retrospectively analysed 87 DLBCL patients who underwent baseline FDG PET/CT and followed the R-CHOP or R-CHOP-like strategy. The clinical parameters and PET-related metabolic parameters were evaluated.

**Results:**

For all patients, the 2-year PFS rate was 65.5% and the 2-year OS rate was 66.7%. According to Cox multivariate analysis, a high NCCNIPI score (4–8 points) and an MTV greater than 64.1 cm^3^ (defined by ROC) were independent prognostic factors for PFS and OS. The patients were divided into low, low-intermediate, high-intermediate and high-risk groups by NCCNIPI score. The 2-year PFS rates in each group were 90.9%, 71.3%, 33.2% and 16.7%, and the 2-year OS rates were 100%, 81.6%, 48.4% and 16.7%. In the subsequent subgroup analysis by MTV, it could further stratified low-intermediate and high-intermediate NCCNIPI groups, the P value was 0.068 and 0.069 for PFS, 0.078 and 0.036 for OS.

**Conclusions:**

MTV, as a tumor metabolic volume parameter, and the NCCNIPI score were independent predictors of prognosis in general DLBCL patients. In the low-intermediate and high-intermediate NCCNIPI subgroup, we further confirm the risk stratification abilities of MTV, which could add the prognostic value of NCCNIPI.

**Electronic supplementary material:**

The online version of this article (10.1007/s12149-020-01531-1) contains supplementary material, which is available to authorized users.

## Introduction

Diffuse large B-cell lymphoma (DLBCL) is the most common type of non-Hodgkin's lymphoma, accounting for approximately one-third of adult cases of non-Hodgkin's lymphoma [1]. In the past 20 years, the long-term remission rate and cure rate of this aggressive lymphoma have been improved by adding rituximab to the standard CHOP (cyclophosphamide, doxorubicin, vincristine, and prednisone) strategy [2]. However, despite the significant improvement, more than 30% of patients receiving R-CHOP or R-CHOP-like chemotherapy have become refractory or have experienced recurrent lymphoma, leading to treatment failure [3]. Therefore, the accurate risk stratification of these patients before treatment and the early detection of patients who are unlikely to be cured are important factors guiding treatment. Since 1993, the International Prognostic Index (IPI) has been used to predict the prognosis of aggressive NHL treated with doxorubicin-based therapy; this index is based on five stratified clinical characteristics: age, Ann Arbor stage, lactate dehydrogenase (LDH), number of extranodal sites, and Eastern Cooperative Oncology Group (ECOG) performance status [4]. However, the IPI was developed before the era of rituximab. After adapting to the new era, the index was refined, and a revised IPI (R-IPI) [5]and NCCNIPI [6] were produced, which improved and refined the risk stratification. However, some refractory and relapsed patients still have not been identified, and the stratified approach based on clinical characteristics and biochemical indicators does not include all the patient's features, such as tumour metabolic burden. In recent years, FDG PET/CT has been widely used and recommended by the International Lymphoma Working Group for lymphoma in terms of pretreatment staging, treatment response evaluation, and prognosis prediction [7]. For DLBCL, many studies have shown that semiquantitative volume parameters such as metabolic tumour volume (MTV) and total metabolic glycolysis (TLG) are independent risk factors for patient prognosis [8–10]. A higher MTV is significantly associated with poor PFS and/or OS. Though the volume parameter shows its prognostic ability in overall DLBCL patients, according to our knowledge, and further stratification by MTV in subgroup has not been confirmed and studied, the aim of our study was to provide evidence to support this point.

## Materials and methods

### Population

A total of 87 consecutive patients (mean age ± SD = 55.8 ± 15.3) were enrolled in our hospital from October 2012 to October 2018. The inclusion criteria were as follows: 1. all patients were older than 18 years; 2. were new cases of lymphoma; 3. all patients were confirmed to have DLBCL by pathology and immunohistochemistry; 4. all patients underwent baseline whole body PET/CT glucose metabolism imaging within 2 weeks before treatment and accepted the R (rituximab)-CHOP (cyclophosphamide + anthracycline + vincristine + prednisone) regimen or R-CHOP-like regimen as a first-line treatment; and 5. the effective follow-up time (censored or no event occurred at the end of follow-up) was more than 24 months. The exclusion criteria were as follows: 1. patients who had central system lymphoma; 2. patients with other types of malignant tumours; and 3. patients who were not treated according to the standardized R-CHOP regimen.

The origin of DLBCL is divided into a GCB (germinal centre B cell) origin and a non-GCB origin according to Hans standards [11]. All patients were scored from 0 to 8 based on age, serum lactate dehydrogenase (LDH) level, Ann Arbor staging, extranodal infiltration and ECOG status according to the NCCNIPI criteria [6].This study was retrospectively reviewed and approved by the ethics committee.

### PET/CT imaging

All images were obtained with a PET/CT scanner (Discovery VCT 64 [GE Healthcare]). 18F-FDG was produced by our hospital, and the radiochemical purity was > 95%. All enrolled patients completed PET/CT scans within 2 weeks before treatment initiation. All patients fasted for more than 6–8 h, and their glucose level were lower than 150 mg/dl. The total activity was administered at 4.4–5.5 MBq/kg per kilogram of body weight. After the injection, the patient was instructed to rest in the supine position for 45–60 min, and then whole-body PET/CT imaging was performed; the image acquisition process used 140 kV, automatic mA, volume imaging, and a reconstruction layer thickness of 3.75 mm; the PET images were collected from six to seven beds according to the height of the patient, and the patient remained breathing peacefully when the images were collected. An ordered subset with attenuation correction was used to maximize the expectations to reconstruct the PET images.

### Image analysis

We imported all PET/CT image sequences into the Fiji software PET plug-in (Beth Israel plug-in for FIJI (ImageJ, Bethesda, MD, USA)) [12]. Positive lesions were defined by visual assessment as limited or diffuse above ambient background uptake lesions. This software has been successfully applied in previous studies for semiquantitative analysis [13]. The target volume (VOI) was then semi-automatically delineated, and it included all the lesions in the axial, sagittal, and coronal planes. The VOI excluded physiological uptake, and then the SUVmax, SUVmean, MTV, and TLG were automatically generated. The MTV automatically calculated the lesion volume of the VOI with the SUVmax = 41% threshold method, which is based on the recommendation of the European Association of Nuclear Medicine [14].Similarly, SUVmean was also automatically calculated and generated. The tumour glycolysis rate (TLG) was calculated as MTV × SUVmean. All calculation results were measured and confirmed by two doctors.

### Statistical analysis and ethics

Statistical analysis was performed using IBM SPSS Statistics (Version 20.0; IBM Corp., New York, USA) software. For the quantitative data, we showed the median and range; tumour progression or death was the end point of follow-up for all patients. We used the Kaplan–Meier method to estimate the survival curve and calculate progression-free survival (PFS) and overall survival (OS). Survival analysis variables included 18F-FDG PET parameters and clinically relevant variables. Receiver operating characteristic curve (ROC) analysis was used to determine the cutoff value of TMTV to predict OS, and continuous variables were divided into two categorical variables based on the cutoff value. Only indicators with an area under the curve (AUC) greater than 0.7 were further analysed. The Spearman rank correlation was used to evaluate the relationship between the MTV and TLG. Because of multiple collinearity, the larger area under the curve was selected for Cox analysis. Univariate and multivariate analyses evaluated the significance of the predictive value. The hypothesis test was two-tailed, and a *p* value of less than 0.05 was considered statistically significant. We still used the k–m method in the subgroup analysis, and *p* < 0.1 were considered statistically significant.

### Follow-up

Follow-up was performed by telephone or the outpatient system. We followed the patients until June 2019, with a median follow-up time of 28 months (1–84 months). PFS was the time from pathologically confirmed DLBCL to the first appearance of progression or the exclusion of other causes of death (months).

## Results

### Patient characteristics and incidence rate of event

The patients’ clinical information and tumour characteristics are summarized in Table 1. At the end of follow-up, events occurred in 30 patients in 2 years, the PFS rate was 65.5% in 2 years, 29 patients died in 2 years, and the 2-year OS rate was 66.7%.

**Table 1:** Characteristics of all patient

Characteristics	Value
Sex	*n* = 87(%)
Female	44(50.6%)
Male	43(49.4%)
Age (years)	
≤ 60	46(52.9%)
> 60	41(47.1%)
Ann Arbor stage	
I	17 (19.5%)
II	26 (29.9)
III	18 (20.7)
IV	26 (29.9)
ECOG status	
0–1	68 (78.2%)
≥ 2	19 (21.8)
LDH level (normal = 245)	
≤ 1	51 (58.6%)
1–3	26 (29.9%)
> 3	10 (11.5%)
NCCNIPI	
Low 0–1	12(13.8%)
L-I 2–3	38(43.7%)
H-I 4–5	31(35.6%)
High ≥ 6	6 (6.9%)
Origin	
GCB	46 (52.9)
NonGCB	41 (47.1)
SUVmax(g/ml)	
Mean (SD)	18.12(8.56)
Median (range)	18(2.6–43.9)
SUVmean(g/ml)	
Mean (SD)	12.16(11.52)
Median (range)	10.8(1.3–106)
MTV(cm3)	
Mean (SD)	176.33(229.76)
Median (range)	78.6(1.8–1156)
TLG(cm3)	
Mean (SD)	2193.09(2875.00)
Median (range)	1014.8(5.2–14, 029.2)

*ECOG* Eastern Cooperative Oncology Group, *LDH* lactate dehydrogenase, *NCCNIPI* National Comprehensive Cancer Network International Prognostic Index, *GCB* germinal centre B cells, *MTV* metabolic tumour volume, *TLG* total metabolic glycolysis

### Baseline PET metabolic parameters and ROC cutoff

The median SUVmax, SUVmean, MTV, and TLG were 18 (range 2.6–43.9), 10.8 (range 1.3–106), 78.6 (range 1.8–1156), and 1014.8 (range 5.2–14,029.2), respectively (Table 1). This study used the 2-year OS rate as the main study end point, making ROC curves for the metabolic indicators, and defined the maximum area cutoff under the curve as the best visualized cutoff. For SUVmax and SUVmean, the areas under the curve were 0.641 (range 0.524–0.759) and 0.615 (range 0.496–0.734), and the *p* values were 0.032 and 0.081. For the MTV and TLG, the area under the curve was 0.742 (range 0.640–0.844) and 0.724 (range 0.620–0.829), and the *p* values were 0.0002 and 0.0007. Since the TLG was calculated by the MTV, there was strong collinearity, and the Pearson correlation coefficient was 0.913, *p* < 0.0001. Therefore, the MTV with a larger area under the curve was selected for multivariate analysis. When the cutoff value was 64.1, the area under the curve was the largest, with sensitivity = 0.897, specificity = 0.603, and Youden index: 0.5 (see Table 2).

**Table 2:** Area under the curve

Parameter	AUC	Standard error	*p* value	95% CI	
				Low	High
SUVmax	0.641	0.060	0.032	0.524	0.759
SUVmean	0.615	0.061	0.081	0.496	0.734
MTV	0.742	0.052	0.000	0.640	0.844
TLG	0.724	0.053	0.001	0.620	0.829

### Univariate and multivariate analyses of prognostic risk factors

Taking PFS and OS as the end points, age, sex, cell origin, extranodal involvement status, ECOG status, serum lactate dehydrogenase level, staging, NCCNIPI score, and MTV > 64.1 cm^3^ were included in the univariate analysis. An age over 60 years, an Ann Arbor stage III–IV, a high NCCNIPI score (4–8 points), and an MTV greater than 64.1 cm^3^ were significantly related to PFS and OS (*p* = 0.03, 0.007, 0.0001, 0.0001, and 0.0131, 0.004, 0.00003, 0.00008, respectively); extranodal involvement status for PFS was statistically significant, *p* = 0.02. Because age, extranodal involvement status and stage are all components of the NCCNIPI, only the NCCNIPI and MTV were included in the Cox multivariate analysis. Both indicators were independent risk factors for PFS and OS: NCCNIPI (PFS: HR, 2.462, 95% CI, 1.184–5.119, *p* = 0.016, and OS: HR2.748, 95% CI 1.309–5.771, *P* = 0.008); MTV > 64.1(PFS: HR, 3.609, 95% CI, 1.492–8.728, *p* = 0.004, and OS: HR3.953, 95% CI 1.561–10.011, *p* = 0.0038), as shown in Table 3 and Table 4.

**Table 3:** Univariate analysis and log-rank test for PFS and OS

Variable	PFS	OS				
	HR	95% CI	*p* value	HR	95% CI	*p* value
Age	1.495	1.036–2.157	0.03145	1.606	1.104–2.335	0.01314
Sex (male)	0.901	0.475–1.708	0.74947	1.101	0.574–2.111	0.77294
Org (non-GCB)	1.382	0.732–2.608	0.31848	1.297	0.680–2.473	0.43032
Extranodal (yes)	2.142	1.109–4.136	0.02332	1.911	0.970–3.763	0.06107
ECOG status (> 2)	1.196	0.567–2.521	0.63898	1.217	0.574–2.581	0.60808
LDHrato			0.26285			0.33165
LDHrato (1)	1.744	0.877–3.467	0.11264	1.495	0.731–3.058	0.27059
LDHrato (2)	1.533	0.606–3.876	0.36699	1.847	0.731–4.669	0.19460
Ann Arbor III-IV	2.564	1.297–5.070	0.00679	2.805	1.384–5.685	0.00422
NCCNIPI (≥ 4)	4.064	2.043–8.082	0.00006	4.477	2.209–9.073	0.00003
MTV > 64.1	5.221	2.294–11.886	0.00008	5.881	2.443–14.162	0.00008

**Table 4:** Multivariate analysis of NCCNIPI and TMTV for PFS and OS

Variable	PFS	OS				
	HR	95% CI	*p* value	HR	95% CI	*p* value
NCCNIPI (≥ 4)	2.462	1.184–5.119	0.01588	2.748	1.309–5.771	0.00756
MTV > 64.1	3.609	1.492–8.728	0.00440	3.953	1.561–10.011	0.00375

### K–M survival curve estimation of independent risk factors and subgroup stratification by MTV cutoff value on L-I and H-I NCCNIPI subgroup

Different NCCNIPI scores are effective for predicting the prognosis of lymphoma patients. The K–M method was used to predict survival curves. The low-risk group (NCCNIPI 0–1 points) had a 2-year PFS and OS rate of 90.9% and 100%; in the low-intermediate group (NCCNIPI 2–3 points), 2-year PFS and OS rate was 71.3% and 81.6%; in the high-intermediate group (NCCNIPI 4–5 points), 2-year PFS and OS rate was 33.2% and 48.4%; and in high-risk group 2-year PFS and OS rate was 16.7% and 16.7%. The *χ*^2^ value was 21.166 for PFS and 24.453 for OS, and the *p* values were 0.0001 and 0.00002 (Supplemental Fig. 1–1; 1–2), respectively.

For the volume parameter MTV, the best cutoff value was 64.1 cm^3^. The high-volume group (MTV > 64.1 cm^3^, *n* = 49) had a 2-year PFS rate of 36.9% and an OS rate of 49%. The low-volume group (MTV ≤ 64.1, *n* = 38) had a 2-year PFS rate of 83.5% and an OS rate of 92.1%. The log rank *χ*^2^ was 19.771 for PFS and 20.194 for OS. The *p* values were 0.000009 and 0.000007 (Supplemental Fig. 2–1; 2–2).

We further stratified L-I risk and H-I risk NCCNIPI subgroups by MTV to confirm the prognostic capability of the metabolic parameter; in the L-I group: NCCNIPI3-4 (*n* = 38), patients with low MTV had better 2-year survival rate (83.6% for PFS and 94.7% for OS) than those with high MTV (59.2% for PFS and 68.4% for OS), and the *P* value was 0.068 and 0.078. In the H-I group: NCCNIPI5-6 (*n* = 31), patients with low MTV had better 2-year survival rate (83.3% for PFS and 100% for OS) than those with high MTV(23.1% for PFS and 36% for OS), and the *P* value was 0.069 and 0.036 (Supplemental Fig. 3–1; 3–2; 3–3; 3–4).

## Discussion

In this article, the tumour burden metabolic parameter and the NCCNIPI score were independent risk factor for the prognosis of DLBCL and helped in the prediction of PFS and OS. The NCCNIPI score is composed of five indicators. In univariate analysis, although ECOG status and LDH level were not significant (*p* > 0.05) due to the small sample size, when the five indicators were combined to form the NCCNIPI score, the prediction efficiency of the score had statistical significance (*P* = 0.00006), which shows that the ability of comprehensive scoring is more convincing than a single indicator for predicting prognosis, avoiding random errors caused by insufficient sample size.

When we divided the patients by NCCNIPI into four subgroup as low(0–1), low-intermediate (2–3), high-intermediate (4–5), and high (6–8) risk group, the survival curve was separated clearly, *p* < 0.0001, showing results similar to those of Zhou et al. [6].

As the cells of origin (COO) are controversial, the Hans (CD10, BCL6, and MUM1/IRF4) method was used to classify the cells of origin (GCB/non-GCB) in our article [11]. In the univariate analysis, there was no statistical significance, which was not enough to confirm the predictive effect on PFS and OS. Previous studies have suggested that the use of immunohistochemistry (IHC) to perform cell origin typing cannot predict patient survival [15, 16], but gene expression profile (GEP)-related technologies such as rapid reverse transcriptase multiplex ligation-dependent probe amplification assay (RT-MLPA) technology [17], DASL (cDNA-mediated annealing, selection, ligation and extension) [18], etc., which classify the origin of tumour cells, have a clear role in predicting the prognosis of DLBCL. Our results also suggest that the clinical immunohistochemical classification cannot be used as an index of patient prognostic risk classification.

In DLBCL, whether the baseline PET imaging characteristic SUVmax is an independent prognostic factor is controversial; early studies have suggested that SUVmax is an independent prognostic factor [19, 20]. In recent years, with the recognition of volume metabolism parameters and the observation of large samples, it has been concluded that volume metabolism parameters can improve the accuracy of DLBCL prediction [9].Regardless of SUVmax and SUVmean, neither the treatment response [21] nor PFS and OS [17] can be predicted.

The baseline PET/CT volume parameter MTV has been confirmed as an independent prognostic factor for PFS and OS in DLBCL in numerous studies [9]. This conclusion was confirmed in a meta-analysis of 13 DLBCL studies [22] and in a recent large-phase III clinical trial (NCT01287741) [23]. In this study, the cutoff value of the MTV was 64.1 cm^3^. The higher metabolic volume group had a worse prognosis than the lower metabolic volume group. The definition of tumour MTV is based on the threshold. The threshold is selected by several methods: SUV ≥ 2.5, SUV ≥ 41% of the SUVmax, and SUV ≥ the average liver uptake background. All methods have similar accuracy in terms of predicting PFS and OS [24].The 41% SUVmax method selected in this study is based on recommendations from the European Association of Nuclear Medicine [14].

The advantage of this article lies on further confirmation of the prognosis value of MTV as the most reliable volume parameter in the subgroup of NCCNIPI system, which excludes the interference of different subgroups determined by clinical indicators on the prognosis stratification. In previous studies [25], a similar subgroup analysis was performed, but it did not strictly follow the NCCNIPI subgroup standard. In our 2 subgroups (L-I risk and H-I risk), MTV also distinguish their prognosis by the 64.1 cm^3^ as the cutoff value of higher volume and lower volume. In the L-I risk group, the *p* values were 0.068 for PFS and 0.078 for OS, and in the H-I risk group the *P* value was 0.069 and 0.036, which were all statistically significant at a *p* < 0.1 level. This is a small sample and retrospective study; when we stratified with a relatively small sample size in the NCCNIPI subgroup according to the MTV parameters and performed survival analysis, we could already get a clear difference in survival, and the two survival curves were separated clearly. Although part of them, the* P* value were not less than 0.05, we thought 0.1 could also illustrated the difference, which have provided ideas and directions for further multi-centre prospective large sample researches.

Recently, many new biochemical molecular markers, such as ctDNA [26], have appeared to predict the prognosis of DLBCL. Image-related metrics also constantly emerge. In the future, the combined prediction of clinical indicators as well as blood biochemical, pathological and functional imaging metrics or radiomics metrics [21] will become a trend.

The limitation of this article is the single-centre retrospective design. A further validation is needed to be carried out in multicentre prospective cohorts.

## Conclusion

The baseline volume metabolic parameter MTV and the NCCNIPI score are independent prognostic risk factors for DLBCL patients treated with R-CHOP or R-CHOP-like regimens. In our study, we further confirmed the risk stratification function of MTV as an FDG metabolic parameter in the NCCNIPI L-I and H-I risk subgroups, which support that physicians make the decision not only using a traditional scoring system, but also by MTV as a supplement for the patients' individual therapy strategy.

## Electronic supplementary material

Below is the link to the electronic supplementary material.

Supplementary file1 (DOCX 586 kb)
