# Supplementary material for: Prognostic value of the baseline 18F-FDG PET/CT metabolic tumour volume (MTV) and further stratification in low-intermediate (L-I) and high-intermediate (H-I) risk NCCNIPI subgroup by MTV in DLBCL MTV predict prognosis in DLBCL
Source: Ann Nucl Med. 2020 Oct 1;35(1):24–30. doi: 10.1007/s12149-020-01531-1 (PMC7796872; doi:10.1007/s12149-020-01531-1)
Supplement: Supplementary file 1 [file 12149_2020_1531_MOESM1_ESM.docx]

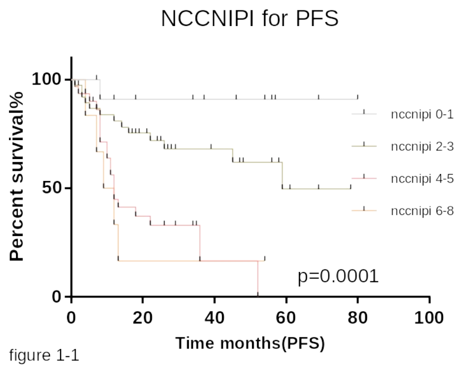

figure 1-1

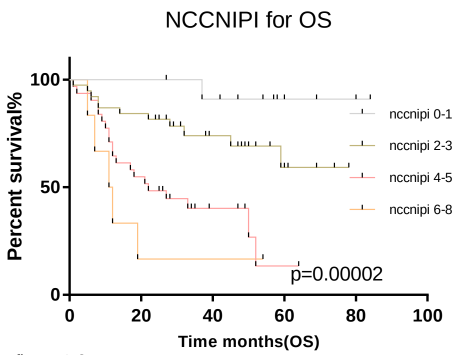

figure 1-2

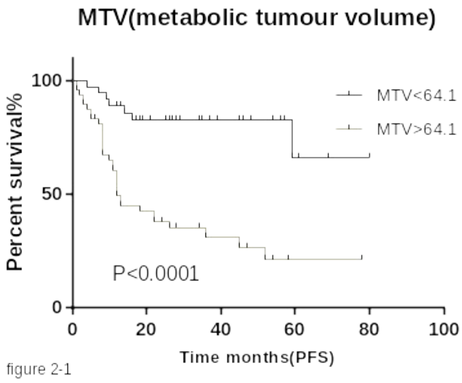

figure 2-1

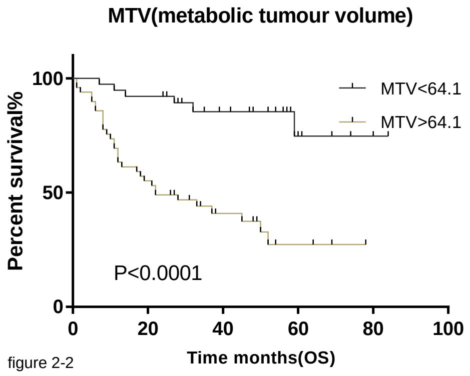

figure 2-2

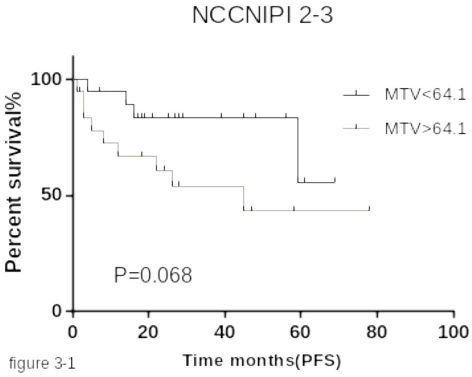

figure 3-1

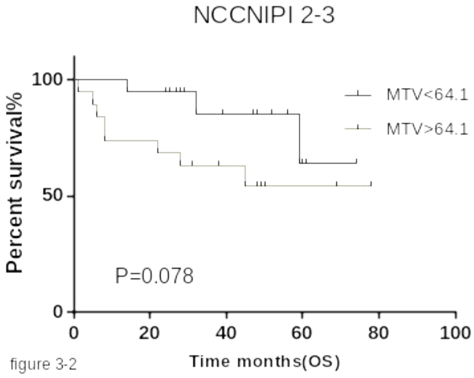

figure 3-2

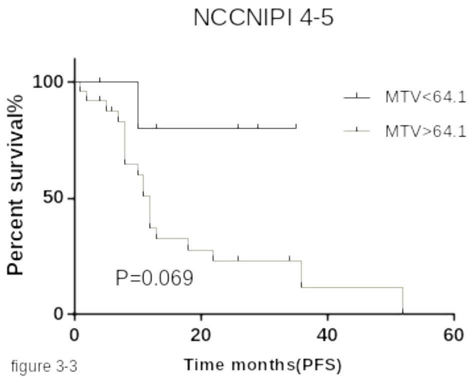

figure 3-3

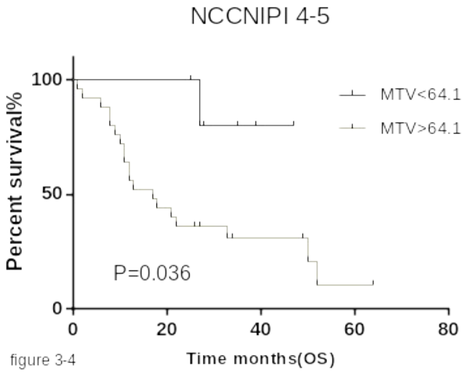

figure 3-4
